# Supplementary figures and images for: Biology of a putative male aggregation-sex pheromone in Sirex noctilio (Hymenoptera: Siricidae)
Source: PLoS One. 2020 Dec 31;15(12):e0244943. doi: 10.1371/journal.pone.0244943 (PMC7775065; doi:10.1371/journal.pone.0244943)

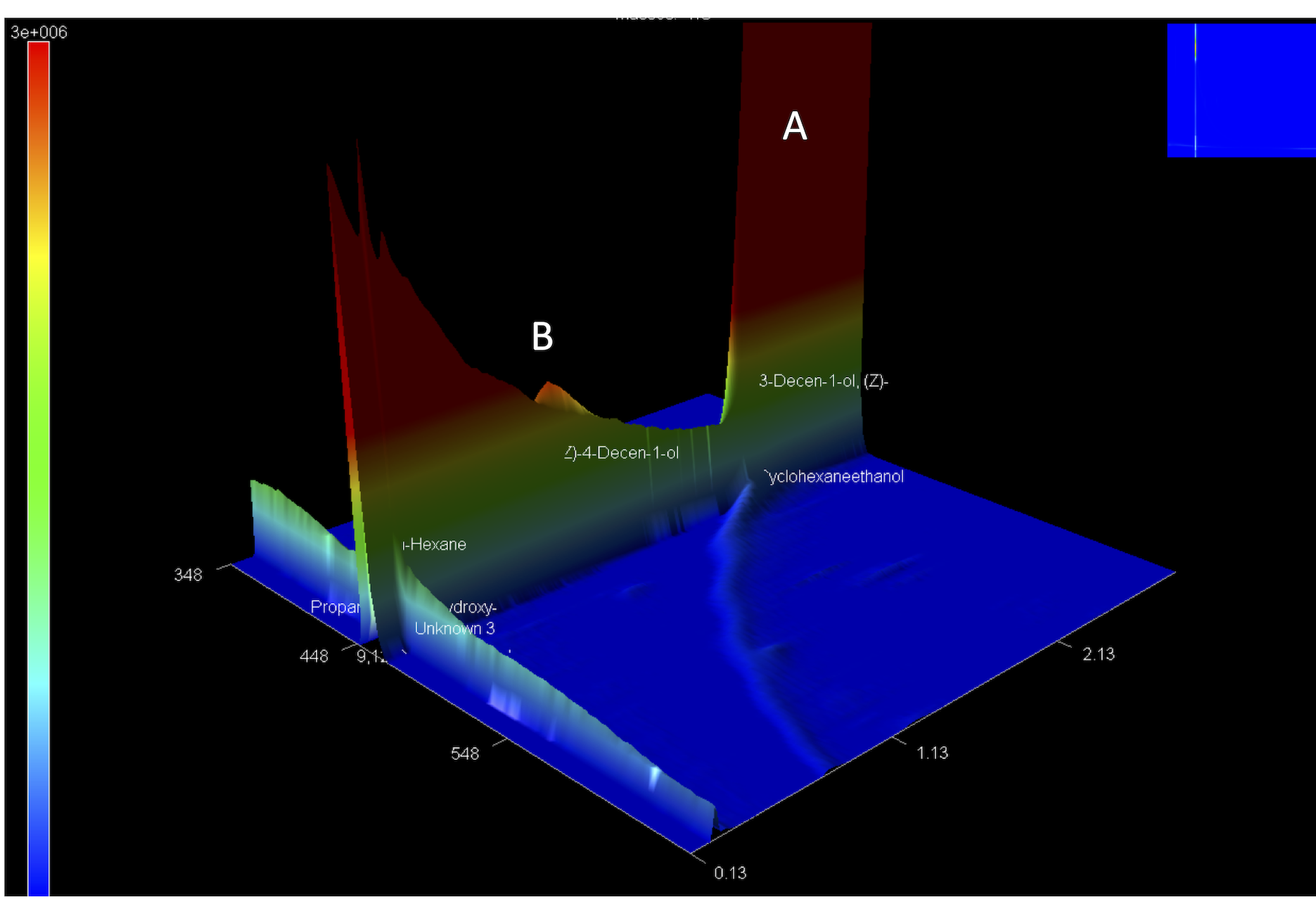

Supplement: S1 Fig — The best tentative match for peak A was (Z)-3-decenol and peak B was (Z)-4-decenol in the NIST library. (TIFF) [file pone.0244943.s001.tiff]

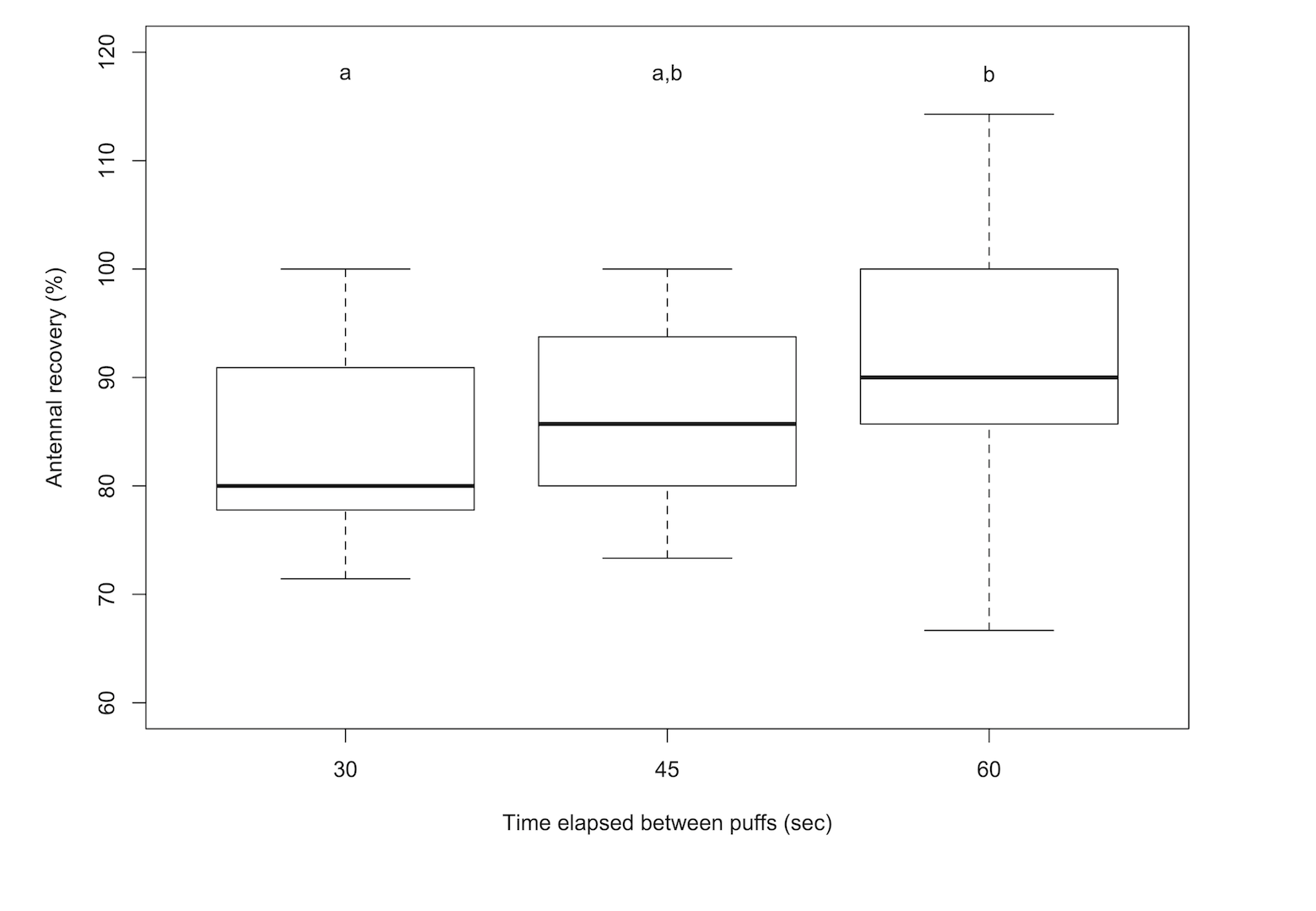

Supplement: S2 Fig — Different letters indicate significant differences of the recovery time. (TIFF) [file pone.0244943.s002.tiff]
