# Supplementary material for: Biology of a putative male aggregation-sex pheromone in Sirex noctilio (Hymenoptera: Siricidae)
Source: PLoS One. 2020 Dec 31;15(12):e0244943. doi: 10.1371/journal.pone.0244943 (PMC7775065; doi:10.1371/journal.pone.0244943)
Supplement: Attachment — Submitted filename: Response to Reviewers.docx [file pone.0244943.s003.docx]

Faculty of Natural & Agricultural Sciences

Department of Zoology and Entomology

Academic Editor

Dr Gregg Roman

PLOS ONE

Dear Dr Roman,

**Submission of Revised Manuscript**

Thank you for your positive feedback regarding our manuscript, entitled “The putative male aggregation-sex pheromone in *Sirex noctilio* (Hymenoptera: Siricidae)” (edited to: “Biology of a putative male aggregation-sex pheromone in *Sirex noctilio* (Hymenoptera: Siricidae)”). We believe that the suggestions made by the reviewers have improved the scientific quality and clarity of our manuscript.

In this revision, we incorporated most of the suggestions made by the reviewers, which has resulted in a number of minor changes to our manuscript. These changes include clarification about the Methods and Materials and more explicit acknowledgement of the limitations of this study. All suggested minor suggestions including spelling, grammar and style have been attended to in the revised manuscript.

Please find attached our revised manuscript. Our response to the comments and suggestions made by the reviewers can be found in the document titled “Response to Reviewers”, a marked up copy of the manuscript can be found in the document titled “Reviewed Manuscript with Track Changes” and the unmarked version of the manuscript can be found in the document titled “Manuscript”. We have also included a copy of figure 3, revised as suggested by reviewer 3.

We thank you in advance for your assistance with this manuscript.

Kindest regards,

Jeremy Allison

**Response to Reviewer Comments and Suggestions**

Please note that the page and line numbers referred to in our responses represent those found in the newly submitted “Manuscript” document.

**Reviewer 1:**

1. **The reviewer asked if commercial lures exists**
   - Commercial lures of the *S. noctilio* male pheromone are not available.
2. **The reviewer asked if we loaded solvent with the pheromone in the red rubber lure**
   - Yes. We clarified how we loaded the red rubber septa with solvent in lines 273-275.
3. **The reviewer asked on what basis our formulations were selected.**
   - The lure was made to mimic previously published work [25, 31]. Although these were high doses it did not match male release rates. This has been clarified in lines 265-267.
4. **The reviewer asked for clarification on the lack of effect of the pheromone blend previously used in field trials**
   - Traps baited with pine volatiles or pine volatiles and the putative male pheromone blend captured similar numbers of female *S. noctilio*. This has been clarified (see lines 75-76).
5. **The reviewer asked if the Teflon tubes were capped**
   - Yes. We added additional text in line 272.
6. **The reviewer asked for clarification on why we stated that the lure previously used likely had a similar release rate to the one used in this study**
   - We have edited this section to state that “If the lures previously tested in the field [25,31] and those sampled in this study have similar release rates, their low release rate might have contributed to the negative results (lines 563-564).
7. **The reviewer suggested that a longer recovery time may have been needed between puffs**
   - This was a trade-off between a decrease in antennal sensitivity over time and the time between puffs of standard. In our set-up, one experiment was conducted over ~8 min. If we would have used two minutes or more between puffs, each experiment on one antenna would have taken ~20 min and antenna were more likely to lose sensitivity/die during this longer time window. We added text to acknowledge this limitation and trade-off (see lines 545-549).
8. **The reviewer asked for more details on the EAG method.**
   - We added additional text to lines 151, 222 and 225-227.
9. **The reviewer asked if table 1 was needed.**
   - We think table 1 adds clarity for the reader. To minimize redundancy, the Kovat indexes were removed from the text.
10. **The reviewer asked to remove the paragraph linking EAD response to odorant receptors.**
    - We agree with the reviewer and we removed the paragraph (lines 499 to 505 in the original manuscript).
11. **The reviewer suggested an article that could be included in the discussion**
    - The suggested article had been included in the discussion lines 508-509.
12. **The reviewer asked if female extractions were identical to blank or to male extracts.**
    - The blank and the female chromatogram were identical in the poropak extraction. This has been observed previously in this species by Cooperband et al. (2012).

**Reviewer 2:**

1. **The reviewer suggested a title change**
   - Title was changed to “Biology of the putative male aggregation-sex pheromone in *Sirex noctilio* (Hymenoptera: Siricidae)”
2. **The reviewer asked to add citations in the introduction**
   - Citation were added in lines 47, 48, 49 and 50.
3. **The reviewer suggested some minor restructuring of the introduction.**
   - We restructured the paragraph according to the reviewers’ suggestion in lines 59-66.
4. **The reviewer requested additional information be added to the first paragraph of the Materials and Methods regarding log collection, insect storage and the dynamic and static headspace sampling**
   - We clarified log collection time, size, number, emergence time and provided more information on insect storage in lines 88-94. We also added additional information regarding the dynamic and static sampling (see lines 106-107, and 117-119).
5. **The reviewer asked what algorithm were used and under which parameter**
   - The original draft included reference to the study that developed the algorithm and approach (line 151). We have added no additional text.
6. **The reviewer asked how was the amount of standard puffed determined and if it was a standard procedure.**
   - We selected a broad spectrum of doses to cover the full spectrum of antennal sensitivity. No additional text has been added.
7. **The reviewer asked what determined if an antennal trial was successful.**
   - When an antennal response was visible for both positive controls puffed, the trial was considered as successful. This clarification was added (lines 216-217).
8. **The reviewer asked why we did a Kruskal-Wallis test.**
   - The data were not normally distributed. This information was added to line 299.
9. **The reviewer asked what is the actual p.value line 432 (p > 0.05).**
   - The differences between males and females were not significant for the six doses of (*Z*)-3-decenol tested. We clarified this in line 432.
10. **The reviewer suggested to remove a paragraph from the discussion (lines 496-505 in the revised draft)**
    - We think that this paragraph builds on our interpretation that these compounds may not be a sex pheromone and have therefore retained this text.
