# Supplementary material for: Biology of a putative male aggregation-sex pheromone in Sirex noctilio (Hymenoptera: Siricidae)
Source: PLoS One. 2020 Dec 31;15(12):e0244943. doi: 10.1371/journal.pone.0244943 (PMC7775065; doi:10.1371/journal.pone.0244943)
Supplement: Attachment — Submitted filename: Response to ReviewersR2.docx [file pone.0244943.s004.docx]

Faculty of Natural & Agricultural Sciences

Department of Zoology and Entomology

Academic Editor

Dr Gregg Roman

PLOS ONE

Dear Dr Roman,

**Submission of Revised Manuscript**

Thank you for your positive feedback regarding our manuscript “Biology of a putative male aggregation-sex pheromone in *Sirex noctilio* (Hymenoptera: Siricidae)”. We believe that the suggestions made by the reviewers have improved the scientific quality and clarity of our manuscript.

In this revision, we incorporated most of the suggestions made by the reviewers, which has resulted in a number of minor changes to our manuscript. These changes include clarification about how the septa lures were loaded in the Materials and Methods, as requested by Reviewer #1. All suggested minor suggestions including spelling, grammar and style have been attended to in the revised manuscript. We thank Reviewer #2 for their suggestions which have improved the clarity of this manuscript.

Please find attached our revised manuscript. Our response to the comments and suggestions made by the reviewers can be found in the document titled “Response to Reviewers”, a marked up copy of the manuscript can be found in the document titled “Reviewed Manuscript with Track Changes” and the unmarked version of the manuscript can be found in the document titled “Manuscript”.

We thank you in advance for your assistance with this manuscript.

Kindest regards,

Jeremy Allison

**Response to Reviewer Comments and Suggestions**

Please note that the page and line numbers referred to in our responses represent those found in the newly submitted “Manuscript” document.

**Reviewer 1:**

1. **The reviewer asked for clarification on how the septa were loaded: how much (Z)-3-decenol (mg) was loaded into what volume (uL) of which solvent (hexane?) and then loaded into the septum?**
   - For the silicone ring and the polyethylene micro-centrifuge tube it would not be possible to load the pheromone in solvent as the reservoir is not large enough. The confusion exists re: the red rubber septum. The reviewer is correct, standard practice is to load the pheromone in solvent into the septum. This was not done here, but rather the septum was soaked in the solvent n-hexane for 48 hours and then immediately after the septum was removed from the solvent neat pheromone was loaded into the septum. As the solvent evaporated from the septum the pheromone would have further penetrated the septum matrix. We clarified L273-276: “Red-rubber septa lures (SEPTA) (Insect science) were soaked in n-hexane for 48 h before use in a sealed jar. The septa were removed from the solvent and the putative pheromone was immediately loaded into each septum and then left in a fume hood for 1 hr to allow the loaded material to be absorbed.”
2. **Minor changes**
   - We subscripted “2” L144.
   - Line 273 now reads “Thermo Fisher”.

**Reviewer 2:**

1. **The reviewer asked to use KI instead of Kovat retention indexes on L181 and 307 of the revised draft.**
   - “Kovat retention indexes” is written in full because it is the beginning of a sentence and as per the suggestion below the sentence on L307 has been deleted.
2. **Minor changes**
   - Line 43: commas added.
   - Line 46: change made as suggested
   - Line 47: commas added.
   - Line 59-60: commas added.
   - Line 60-61: commas added and “to” deleted.
   - Line 68-69: Now reads “After emerging from the tree, males form leks in the canopy and females must locate these leks for mating although females are parthogenetic and can produce male progeny without mating [15,28].”
   - Line 80: Now reads “Specifically, we: i) collected…”
   - Line 237: comma added.
   - Line 254: Sirex has been italicized
   - Line 288: change made
   - Line 298: Citation added
   - Line 299: Agreed. The sentence starting on line 300 has been moved to start on line 299 and be part of the preceding paragraph.
   - Line 307-308: Agreed, line 307 now reads “elicit antennal responses (Fig 1, Table 1).”
   - Line 334-335: Done.
   - Line 339-342: we are not sure what reviewer #2 means here and no change has been made.
   - Line 368: Agreed, “is” has been changed to “was”
   - Line 371: Agreed, now reads “…on the second dimension for peaks A…”
   - Line 379: We disagree and have not changed “is” to “was” here.
   - Line 402: Agreed “is” has been changed to “was”
   - Line 475-476: Agreed, SE now in ().
   - Line 485: comma added
